# Dr. Cazenave on Rheumatism

**Published:** 1828-04-01

**Authors:** 


### VIII.

*Memoir on the Treatment of Rheumatism.* By Dr. CAZENAVE, of Pau.

PAU, the town where our author resides, is only six leagues from the Pyrennees. The proximity of these mountains, and the prevailing westerly winds which blow over them, cause most abrupt and extensive transitions of temperature. The country, however, is extremely healthy, with the exception of rheumatism, which may be said to be there endemic. It simulates, like hysteria, almost every other complaint—or complicates itself with almost all other pathological conditions. In such a situation, an observant physician has excellent opportunities of studying a disease, which, after all that has been written on it, is still as obscure in its nature as it is rebellious to treatment. During a period of eight years Dr. Cazenave has cultivated the study of rheumatism in all its ostensible and masked forms, and now comes before his brethren with a method of treatment which he considers as unusually successful.

Unlike some of our modern monographists, Dr. C. has not constructed a volume out of preceding writers:—he has merely made a few remarks, the result of his own observation. We shall extract a passage or two from this Essay, before we state the Doctor's methodus medendi.

"I have seen (says he) rheumatic patients suddenly seized with violent pain in some part of the body, and over a considerable space. On examining with their hands to find out the precise spot, they would have much difficulty in determining it, however hard they might press. At length, a single point, as it were, would be found to be the seat of exquisite pain and sensibility, without any thing being visible externally. In other cases the pain will suddenly cease in a part, and the patient will experience a disagreeable sensation of formication rather than distinct pain in some other part, at a considerable distance. On examination, he is surprised to find a large patch of redness, with more or less tumefaction, in the new seat of the rheumatism. In this manner the complaint will sometimes travel over the whole surface of the body, and then fix itself at the extremity of a finger for a month or two, causing dreadful pain, but without the least discolouration or swelling of the part.

"I have seen this disease in the person of a medical gentleman, fix itself in the sclerotic coat of the eyes, at their outer and inner angles, and there occasion the most terrible sufferings, on the least motion of the eye-ball. Yet the sight was not in the least affected, nor was there any appearance of inflammation. At other times the rheumatalgia has seated itself in the transparent cornea, attended with indescribable sufferings, intolerance of light, insomnium, and violent ophthalmia. The same medical gentleman experiences occasionally the rheumatic pain in a single point of the minutest dimensions, in the eye-brow, and other parts of the face. An elderly lady, afflicted for several years with rheumatalgia of the deltoid muscle, lost slowly her sight. An ophthalmic surgeon discovered a cataract in each eye. The operation was performed on one of them; but scarcely was it finished, when the organ became the seat of the most excessive pain—the sclerotic became gorged—the coats red—and this state of insupportable sufferings lasted six weeks, with scarcely any intermissions. At this period, the deltoid muscle (which had been free from pain) became again the seat of rheumatism, and the ophthalmic inflammation and pain disappeared. She recovered sight in this eye. Three months afterwards the other eye was operated on, and again the pain and ophthalmia took place as violent as in the other eye. A blister was applied to the arm, and a metastasis of pain was quickly produced, and the eye relieved."

Of all the INTERNAL organs, our author has found the STOMACH the most liable to rheumatic affection. In some cases, this shews itself merely in languor of function, or simply a sense of cold or pain in the epigastric region, relieved by hot frictions. The digestion may be very little impeded. In this state of chronicity, the disease is difficult of removal. But, not unfrequently, it produces in the same organ much more disagreeable effects, as nausea, vomitings, indigestion, violent cardialgia, and symptoms imitating cancer or scirrhus of the pylorus—all which phenomena will suddenly disappear on the commencement of rheumatic pain in some of the limbs.

"I have seen a case of wandering rheumatism, where, after attacking the stomach, the bowels, &c. it fixed itself, for more than three months, on the heart, inducing palpitations, convulsions, syncope, and other symptoms that led the attendant physician to believe there was aneurism of the heart. A blister applied to the arm dissipated the whole of these symptoms, and the patient afterwards enjoyed good health."

The author has seen the bladder affected with rheumatism, and retention of urine produced—the lungs attacked, and all the phenomena of peripneumony succeed. Parturient women are very susceptible of the causes of rheumatism, and Dr. C. avers that nothing is more common, at least in his part of the country, than rheumatic pains in the uterus and its appendices.

But we must now proceed at once to the treatment which Dr. C. has brought forward, as it is somewhat novel—at least it is a new modification of a remedy which has long been employed, though less so in this country than formerly. It is opium. After remarking on the different effects of opium, according to the dose, or the repetitions of the doses, Dr. Cazenave proceeds to maintain, that the failure of opium in the cure of rheumatism is owing to the timidity with which it is administered. In the complaint under consideration, Dr. C. remarks, opium acts in three ways, according to the dose employed. Given in small quantities, it obtunds the sensibility, and brings a temporary relief—but the cure is not thereby accelerated. Administered in a somewhat larger dose, it sometimes occasions nausea, palpitations, giddiness, head-ache, &c. These effects are, of course, but momentary, and should form no solid objection to the remedy, if it is found beneficial in other respects, besides relieving pain. To the above effects of opium (if it be continued) succeed others:—the patient does not sleep; but he experiences a kind of delightful ecstacy, forgets his sufferings, &c. The action of opium is then excitant, like that of wine. In some cases, an abundant perspiration is the result—but, in both events, the radical cure of the rheumatism is effected—that is, with or without the sweating process. The quantity of opium will vary, of course, in different constitutions; but the following is the mode of administration employed by our author.

To an adult, he orders a pill containing one grain of opium—and, an hour afterwards, he gives another grain, if the pains continue. At the expiration of the second hour he gives a third grain—and, after a little time, he examines his patient. If there be a tendency to hilarity, he administers a fourth grain, and so on, a grain every hour, till a complete calm is established, or an abundant perspiration is induced. This being the case, he orders a grain to be given every two, three, or four hours, according to circumstances, solely with the view of keeping up the perspiration.

In respect to regimen during this mode of treatment, it is indispensible, of course, to keep the patient in an even and mild temperature, with flannel next the skin, and on the simplest liquid food. Perfect quietude is necessary during this treatment. In this way, Dr. C. assures us that he speedily cures rheumatism, whether acute or chronic, or in whatever part of the body it may be seated, without any bad consequences ever ensuing. When the disease is complicated with any other complaint, particularly with derangement of the digestive organs, it will be necessary to attend to the adventitious disorder. If the fever in acute rheumatism run very high—and particularly if any thoracic or abdominal organ be oppressed in function, or labouring under pain, it will be proper to draw blood from the general system, and to put in force the other items of the antiphlogistic treatment.

We think the plan of Dr. Cazenave is not unworthy of attention, in the management of a disease which so often baffles the medical practitioner, and brings no small degree of odium on his art.

